# Microbiome in connective tissue diseases associated interstitial lung disease

**DOI:** 10.3389/fmed.2026.1738689

**Published:** 2026-03-20

**Authors:** Francesco Puppo, Roberto G. Carbone

**Affiliations:** Department of Internal Medicine, University of Genoa, Genoa, Italy

**Keywords:** connective tissue diseases, dysbiosis, idiopathic pulmonary fibrosis, interstitial lung diseases, microbiome, non-specific idiopathic pneumonia

## Abstract

Microbiome consists of a large community of bacteria, yeast, protozoa, and viruses that co-exist in symbiosis with human hosts. Changes in microbiome, named “dysbiosis,” alter the interplay between microbiome and immune system triggering inflammation and contributing to the pathogenesis of connective tissue diseases (CTD). Interstitial lung diseases (ILD) are a group of pulmonary disorders characterized by lung tissue fibrosis and impaired lung function. The existence of a gut-lung axis is well demonstrated; however, it is not established whether gastrointestinal dysbiosis contributes to ILD development. ILD represent a major cause of morbidity and mortality in CTD patients. Lung microbiome changes and high microbial load are associated with worse prognosis and acute exacerbations in patients with CTD-ILD and especially in those affected by rheumatoid arthritis, systemic sclerosis and dermatomyositis. Probiotics are active microorganisms that normalize the intestinal flora and their use has been proposed as potential supportive treatment of CTD-ILD. Present knowledge of the relationships between dysbiosis and CTD-ILD development is largely incomplete and further studies are needed to validate this issue. Aim of this concise review is to report current knowledge on microbiome in CTD-ILD focusing on clinical lung aspects and therapeutic options.

## Introduction

Microbiome, comprises bacteria, yeast, protozoa, and virus in the cavities and surfaces of human body and consists of trillions of organisms that co-exist in symbiosis with their human hosts. Microbiome interacts with human immune system in a mutual crosstalk and plays a key role in maintaining immune homeostasis through chemical signaling performed by microbiota-derived metabolites (1–3).

Qualitative and/or quantitative changes in microbiome composition, named “dysbiosis,” may alter the interplay between microbiome and immune system triggering inflammation and potentially contributing to the pathogenesis of inflammatory diseases including connective tissue diseases (CTD) (4–7).

Connective tissue diseases are a group of systemic diseases determined by genetic and environmental factors resulting in loss of immunological tolerance that leads to autoreactive immune responses and organ damage. The hypothesis that microbes may play an etiologic role in CTD development has been proposed for many years, however the association between microbial agents and autoimmune diseases has not been definitively established. Environmental and lifestyle changes occurring in the last century contributed to dysbiosis (5). The link between dysbiosis and CTD is increasingly supported by the development of technologies that allow better microbiome characterization and potential correlation with CTD development (8).

Interstitial lung diseases (ILD) comprise a group of pulmonary disorders characterized by the involvement of pulmonary interstitium leading to impaired lung function. ILD may be caused by environmental exposure to trigger agents, like asbestos and allergens, or be associated with CTD. However, the cause of ILD remains in many cases unknown. The most common ILD are idiopathic pulmonary fibrosis (IPF) characterized by progressive interstitial collagen deposition resulting in traction bronchiectasis, reticulation, and honeycomb formation and non-specific interstitial pneumonia (NSIP) characterized by ground glass opacities, traction bronchiectasis and reticular pattern. ILD represent a major cause of morbidity and mortality in patients with CTD.

Recent evidence suggests that lung microbiome changes and high microbial load are associated with worse prognosis and acute exacerbations in patients with ILD, particularly in IPF patients. This latter finding may be related to the presence of traction bronchiectasis with reduced mucoid clearance and infection susceptibility. Moreover, several studies showed that lung microbiome in IPF differs from other ILD and that the immune system may play a role in modifying lung microbiome in CTD-associated ILD (9, 10).

The aim of this concise review is to report current knowledge on microbiome in CTD-ILD focusing on clinical lung aspects and therapeutic options.

## The lung microbiome

The lung is the largest human organ in direct contact with the environment. Microorganisms may penetrate in lungs through microbial inspiration from inhaled air, oral cavities, and gastric content. Microbial intake and growth may be facilitated by defective mucociliary clearance and cough reflex as well as by reduced oxygen partial pressure. Experiments using gene techniques demonstrated that bacteria are present in human lungs and that microbiome changes correlate with modifications in alveolar immunity and lung disease development.

The lung microbiota has a low density than gastrointestinal microbiome and upper airways have a higher abundance and biodiversity than distal airways (10, 11).

In healthy subjects bacterial composition and burden of lung microbiome are similar among individuals and remains constant between various geographic regions. The most prevalent phyla in normal lung comprise Bacteroidetes, Firmicutes, Proteobacteria and Actinobacteria and the most frequent genera are *Prevotella*, *Streptococcus*, *Veronococcus*, *Haemophilus* and *Veillonella* (9).

### The lung microbiome in interstitial lung disease

Most of the past work on microbiome in lung disease focused on chronic obstructive pulmonary disease (COPD), cystic fibrosis and bronchiectasis. More recent research explored lung microbiome in ILD, especially in IPF. In this disease microbiome burden is increased and shows less microbial diversity correlating with disease severity, progression and mortality (12).

**FIGURE 1:**
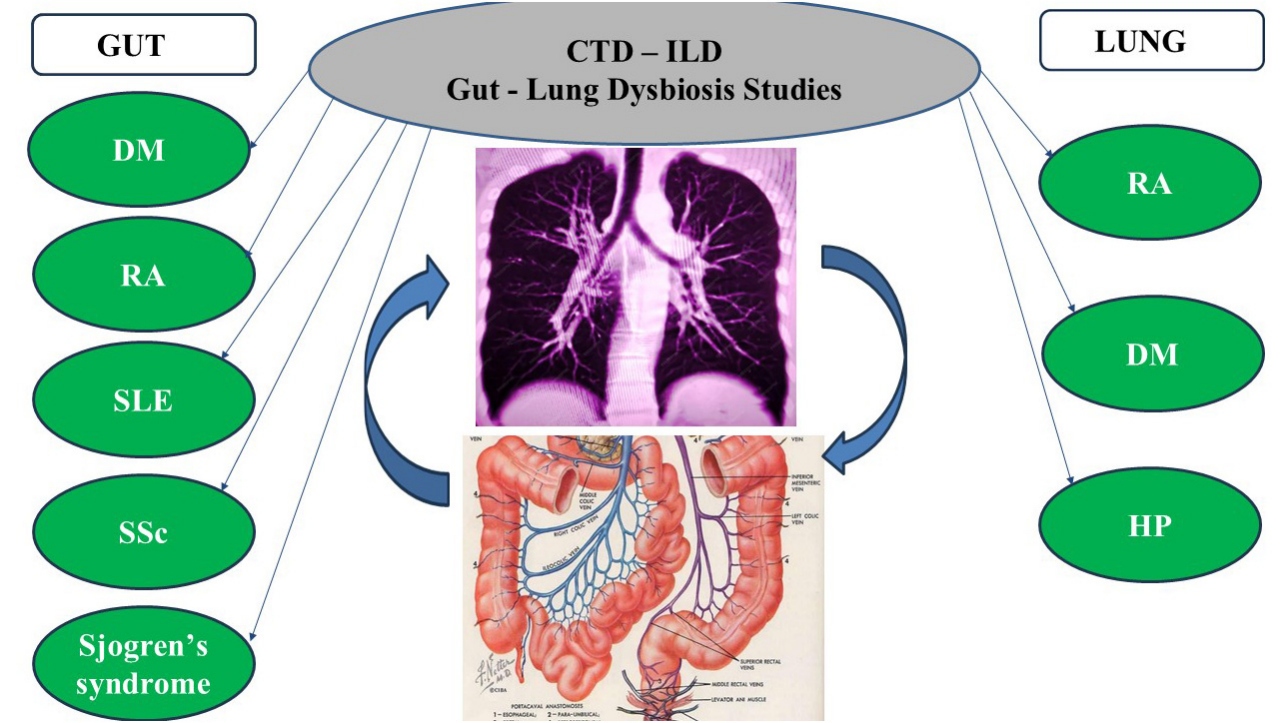
The Figure reports dysbiosis in specific CTD-ILD based on published literature. Curved arrows depict the interactions between gastrointestinal tract and lung microbiota (gut-lung axis). DM, dermatomyositis; RA, rheumatoid arthritis; SLE, systemic lupus erythematosus; SSc, systemic sclerosis; HP, hypersensitivity pneumonia.

The mechanisms by which lung dysbiosis contribute to lung fibrosis are not completely established. It has been suggested that local dysbiosis may stimulate inflammatory immune cells, like T-helper 17 cells, triggering pro-fibrotic pathways (13–19). Gut and lung microbiota changes in CTD are reported in Figure 1 and Table 1.

**TABLE 1:** Microbiota changes in CTD-ILD.

Disease	Gut	Lung
Rheumatoid arthritis	↑ *Porphyromonas gingivalis* ↑ *Aggregatibacter actinomycetemcomitans* ↑ *Leptotrichia* species ↑ *Prevotella copri* ↑ *Lactobacillus*	↑ *Micrococcus* ↑ *Pseudonocardia* ↑ *Renibaterium* ↓ Paraprevotellaceae ↓ Burkholderiaceae ↓ Actinomycetaceae ↓ Spirochaetaceae ↓ *Porphyromonas* ↓ *Treponema*
Systemic sclerosis	↑ Bacteroidetes/Firmicutes ↑ *Desulfovibrio* ↑ Ruminococcus ↑ *Prevotella* ↓ *Faecalibacterium* ↓ *Clostridium*	No studies available
Sjögren’s syndrome	↑ Firmicutes/Proteobacteria ↑ *Lactobacillus* (saliva) ↑ *Streptococcus* (saliva) ↑ Proteobacteria (tears) ↑ *Acinetobacter* (tears) ↑Actinobacteria (tears) ↓ *Corynebacteria* (tears)	No studies available
Systemic lupus erythematosus	↑ Proteobacteria ↑ *Rhodococcus* ↑ *Enterococcus gallinarum* ↑ *Eggerthella* ↑ *Klebsiella* ↑ *Prevotella* ↑ *Eubacterium* ↑ *Flavonifractor* ↑ Ruminococcaceae ↑ *Incertae sedis* ↓ Firmicutes ↓ Dialister ↓ Pseudobutyrivibrio	No studies available
Dermatomyositis	↑ Bacteroides/Firmicutes ↑ Proteobacteria ↓ Christensenellaceae ↓ Ruminococcaceae	↑ Bacteroides/Firmicutes ↑ *Streptococcus* ↑ *Sediminibacterium* ↑ *Veilonella* ↑ *Corynebacterium* ↑ *Aeromonas* ↑ *Achromobacter* ↑ *Pseudomonas*

↑ Indicates increased and ↓ Indicates decreased compared to healthy individuals. For details see text.

## The gut microbiome and the gut-lung axis

The composition of gut microbiome is similar and stable in healthy individuals. Gut microbiome includes mainly Bacteroidetes and Firmicutes. Pulmonary diseases, including chronic obstructive pulmonary disease (COPD), asthma, cystic fibrosis and ILD are associated with alterations in gut microbiome composition (20, 21).

The existence of a gut-lung axis is supported by anatomic communications and common pathways. Alterations of gut microbiota modulate immune responses in the lung including toll-like receptors (TLRs), regulatory T cells (Tregs), and inflammatory cytokines. Interestingly, gut microbiota dysbiosis may be involved in CTD development. Overall, a decrease in microbiome was found in rheumatoid arthritis and systemic lupus erythematosus while an increase was detected in systemic sclerosis (22). Moreover, depletion of anti-inflammatory microbes like *Faecalibacterium* and enrichment of pro-inflammatory microbes like *Streptococcus* was observed in rheumatoid arthritis, Sjögren’s syndrome and systemic lupus erythematosus (23, 24). However, it remains to be established whether GI dysbiosis and GI inflammatory and immunomodulatory molecules contribute to lung disease development.

## Rheumatoid arthritis

Rheumatoid arthritis (RA) is an immune mediated chronic disease characterized by synovial inflammation and joint erosion as well as by internal organ involvement including lungs, pleura, heart, pericardium and eyes. Pleuropulmonary manifestations of RA include ILD, organizing pneumonia, lung nodules, bronchiolitis, bronchiectasis and pleural effusions. ILD is detected in up to 10% of RA patients and is associated with increased morbidity and mortality.

Bacterial dysbiosis in periodontium, lungs, gut and genitourinary system has been suggested as potential trigger for RA initiation (25, 26). Candidate pathogens include *Porphyromonas gingivalis*, *Aggregatibacter actinomycetemcomitans* and *Leptotrichia* species. Interestingly, some bacterial groups, like *Prevotella copri*, were more abundant in early disease whereas other groups, like *Lactobacillus*, were present in increased amounts in active disease (25, 27).

Little is known about the lung microbiome in RA. Available data indicate that bronchoalveolar lavage fluid (BAL) microbiota of RA patients is less varied and abundant in comparison with healthy controls. Paraprevotellaceae, Burkholderiaceae, Actinomycetaceae and Spirochaetaceae were less frequently present in BAL samples from RA patients. In addition, *Porphyromonas* and *Treponema* genus were isolated only in low percentages of BAL from RA patients in comparison with healthy controls. Of interest, *Micrococcus*, *Pseudonocardia* and *Renibaterium* positively correlated with disease activity and *Prevotella* genus correlated with rheumatoid factor IgA and anti-citrulline peptide antibodies levels (26).

## Systemic sclerosis

Systemic sclerosis (SSc) is an immune-mediated disease characterized by vasculopathy and fibrosis of the skin and internal organs, including gastrointestinal tract, skin and lungs. Pulmonary manifestations of SSc include ILD, organizing pneumonia, and isolated pulmonary vascular disease. Asymptomatic ILD can be present in up to 90% of SSc patients although it may be clinically relevant only in 25% of patients. ILD represents the major cause of morbidity and mortality in SSc.

Microbiome dysbiosis might be involved in SSc pathogenesis and, in turn, fibrotic changes in mucosal surfaces can lead to microbiome dysbiosis. Immune cells activated by bacteria may induce endothelial cells damage, neuropathy, myopathy and vasculopathy. Furthermore, Th2 CD4+ T cells infiltrating gastrointestinal mucosa may activate fibroblasts favoring perivascular and interstitial fibrosis (28).

In early SSc, gut microbial dysbiosis is present with increased pathobiont genera like *Desulfovibrio* and *Ruminococcus* and decreased commensal genera like *Faecalibacterium*. SSc patients with advanced disease and gastrointestinal involvement show an increase in *Prevotella* and a decrease in *Clostridium* populations. Of interest, *Clostridium* determines Treg/Th17 cells imbalance that might be responsible for a pro-inflammatory state (29, 30).

Only a few studies have examined the pathogenetic association of gut microbiome and SSc-ILD. A possible hypothesis is that esophageal dysmotility might favor the aspiration of microbes into the lung inducing inflammatory and fibrotic responses. Another potential mechanism might involve immune cells primed by microbes in the gut that reach circulation and modulate fibrotic responses in distant organs including lungs. Notably, patients with SSc-ILD have increased dysbiosis compared with SSc patients without ILD. Fecal calprotectin levels are an indirect marker of gut microbiome dysbiosis and strongly correlate with SSc-ILD progression (31).

Studies in animal models suggest that a shift toward a higher Bacteroidetes/Firmicutes ratio in the gut microbiome is associated with progressive lung fibrosis. Reconstitution of normal gut microbiome, especially with butyrate producing bacteria, downregulates the release of proinflammatory cytokines and ameliorates skin and lung fibrosis suggesting a potential role of butyrate in SSc treatment (30, 31).

**FIGURE 2:**
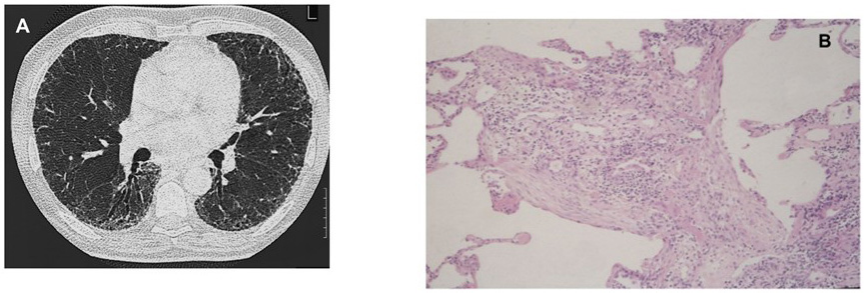
Images of SSc-ILD showing HRCT scan **(A)** and histological **(B)** IPF-UIP pattern. Magnification: H&E ×100.

**FIGURE 3:**
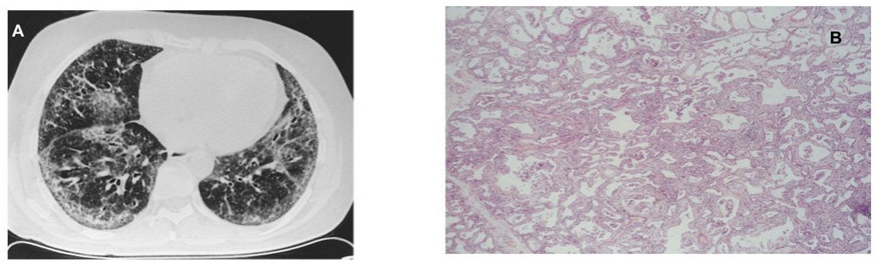
Images of SSc-ILD showing HRCT scan **(A)** and histological **(B)** NSIP pattern. Magnification: H&E ×100.

Interstitial lung diseases are a main cause of SSc-related mortality. However, no studies have specifically evaluated the potential association between lung dysbiosis and ILD development in SSc patients. Preliminary data from a genomic study analyzing gastrointestinal microbiota in SSc demonstrated significant differences in microbiota species between patients with and without ILD correlating with lung disease severity measured by high resolution computed tomography (HRCT) (32). Survival is significantly shorter in SSc patients showing idiopathic pulmonary fibrosis-usual interstitial pneumonia (IPF-UIP) pattern (Figure 2A) than the more common non-specific interstitial pneumonia (NSIP) pattern (Figure 3A) in radiological imaging. UIP-SSc and NSIP-SSc histological findings are reported in Figures 2B, 3B, respectively.

The hypothesis that lung dysbiosis may associate with ILD is supported by the known unbalanced microbial composition in BAL from IPF patients who show an increase in Firmicutes, *Streptococcus*, and *Veillonella* and a decrease in Proteobacteria correlating with alveolar inflammation and disease progression (28).

## Sjögren’s syndrome

Sjögren’s syndrome is an autoimmune disorder characterized by lymphocytic infiltration of the lacrimal and salivary glands with variable involvement of internal organs. Lung involvement consists of lymphocytic infiltration resulting in tracheal-bronchial disease or ILD.

Decreased secretion of salivary and lacrimal fluids that contain antimicrobial factors and alteration of mucosal barriers may increase the risk of dysbiosis development. Antigenic mimicry between bacteria and human cells has been proposed as a potential mechanism underlying microbiome involvement in disease induction. Gut microbiota of Sjögren’s syndrome patients shows a lower Firmicutes/Bacteroidetes ratio in comparison with healthy controls suggesting that the severity of ocular and systemic features may associate with intestinal dysbiosis (33). Contradictory results have been reported on the microbial composition of the buccal mucosa. Some studies indicate that buccal microbiome of patients with Sjogren’s syndrome shows a high Firmicutes/Proteobacteria ratio and does not differ from patients with other sicca syndromes and healthy controls. By contrast, other studies indicate a salivary abundance of *Lactobacillus* and *Streptococcus* in patients with Sjögren’s syndrome as compared to patients with other sicca syndromes (34). Studies on the ocular surface microbiota in subjects with Sjögren’s syndrome indicate that Proteobacteria, *Acinetobacter* and Actinobacteria are the predominant microorganisms whereas *Corynebacteria* are less detected (4–7). Unfortunately, specific studies on the role of microbiome dysbiosis on the development of ILD in Sjögren’s syndrome are lacking.

## Systemic lupus erythematosus

Systemic lupus erythematosus (SLE) is a systemic autoimmune disease caused by a complex interplay of genes, environmental factors, immune responses, and hormones. Pleuropulmonary manifestations are common in about 50%–70% of SLE patients and include ILD, organizing pneumonia, diffuse alveolar hemorrhage, pulmonary hypertension, shrinking lung syndrome, and pleural effusions.

Changes in intestinal barrier, called “leaky gut,” can determine bacteria translocation to the lymph nodes triggering autoimmune phenomena. However, it must be considered that immunosuppressive treatments may affect gut microbiome increasing the risk of dysbiosis.

Gut microbiome dysbiosis has been demonstrated in SLE patients. The genera significantly more represented are Proteobacteria, *Rhodococcus*, *Eggerthella*, *Klebsiella*, *Prevotella*, *Eubacterium*, *Flavonifractor*, Ruminococcaceae, and *Incertae sedis* whereas Firmicutes, *Dialister*, and *Pseudobutyrivibrio* genera are reduced (35–37).

The imbalanced Firmicutes/Bacteroidetes ratio detected in SLE seems to induce immune derangement, in particular a shift to a Th17 activation, responsible of bowel disease. Moreover, the intestinal overgrowth of *Ruminococcus gnavus* and *Enterococcus gallinarum* seems to correlate with disease activity, anti-ds-DNA, anti-Sm and anti- ribosomal P antibodies levels, as well as with development of lupus nephritis (35–37).

No studies have been published on the association between gut dysbiosis and lung disease in SLE patients.

## Dermatomyositis

Dermatomyositis (DM) is an inflammatory myopathy characterized by skeletal muscle and skin inflammation. ILD is the major systemic manifestation associated with DM leading to increased morbidity and mortality.

Patients with DM-ILD have lower microbial diversity and a distinct taxonomic composition compared with healthy controls. The most common genera detected in BAL include *Streptococcus*, *Sediminibacterium* and *Veilonella* especially in patients with positivity for Jo-1 antibodies (12). The amount of microbiota operational taxonomic units (OTUs) detected in BAL of patients with DM-ILD is higher than in BAL of healthy controls. BAL bacterial profiles include predominance of *Corynebacterium*, *Aeromonas* and *Achromobacter*. Zhang et al. (24) demonstrated a significant abundance of *Pseudomonas* and *Corynebacterium* in BAL of DM-ILD. This finding is of particular interest as these species are associated with the production of pro-inflammatory cytokines like IL-2 and IL-8 correlating with disease severity and progression.

Gut microbiome shows a significant increased Bacteroides/Firmicutes ratio inversely correlating with disease severity. Patients with DM-ILD have a reduction of Christensenellaceae and Ruminococcaceae and a significant expansion of Proteobacteria (38). This latter finding is of particular interest as Proteobacteria are a source of bacterial lipopolysaccharide (LPS), a proinflammatory endotoxin causing endothelial damage, oxidative stress, and metabolic dysfunction. Bacteroidetes/Firmicutes ratio is also significantly higher in lung of DM-ILD patients compared to healthy controls. Further studies are needed to validate the correlation between lung dysbiosis and DM-ILD (38, 39).

### Therapeutic approaches

Traditional treatments improve symptoms and prolong survival rates of CTD patients. However, long-term use of glucocorticoids, immunosuppressants and biologics will determine several adverse side effects. Therefore, alternative therapeutic approaches are required to ameliorate CTD treatment. Probiotics are active microorganisms that can colonize the host intestine normalizing the composition of the intestinal flora as well as their metabolism and immune modulating properties. Probiotics may contribute to CTD treatment by correcting intestinal flora imbalance, inhibiting bacteria translocation, regulating CD4+ T cell differentiation, and inhibiting the production of pro-inflammatory molecules.

In this field we report the effect of pro-, pre-biotics and symbiotics on CTD patients. The rationale for probiotics administration is based on gut-lung axis supporting an essential role of gut microbiome in the regulation of lung metabolic, immune and structural mechanisms in health and disease (40). Several investigations show that gut dysbiosis is closely associated with progression of various airways diseases such as asthma, COPD, ILD, and acute lung damage (41–43). The target for probiotic therapy is to repair gut microbiome with the scope of respiratory disease treatment. In this context, a randomized controlled pilot study showed that probiotic (Pro-Vi 5) therapy led to increased quality of life and immune responses modulation in severe COVID-19 infection (44). In pre-clinical studies in a murine model of asthma-COPD overlap syndrome, *Lactobacillus rhamnosus* ameliorated gut dysbiosis decreasing lung inflammation (45). In addition, *Lactobacillus reuteri* reduced lipopolysaccharide-induced acute lung injury in mice through gut microbiome modulation (46). Probiotics most used to treat a variety of gastrointestinal diseases are *Lactobacillus*, *Bifidobacterium* and *Saccharomyces boulardii*. Currently, there is no consensus on clinical use of probiotics in the treatment of CTD (16, 27, 47). A recent meta-analysis of 80 randomized clinical trials evaluating probiotic efficacy in 14 different autoimmune disease showed that gut microbiota-based therapies may improve symptoms and/or inflammatory status of SLE, juvenile rheumatoid arthritis, Sjögren’s syndrome and systemic sclerosis but not of rheumatoid arthritis (48). Therefore, gut microbiota-based therapies may have potential usefulness in the treatment of some CTD. However, further studies are required to definitively confirm the efficacy of probiotics as therapeutic tools for CTD.

Emerging research suggests that high-fiber diets (fruits, vegetables, whole grains) play a protective role in CTD-ILD by gut-lung axis modulation (49). Li et al. (49) showed that dietary fibers, fermented by gut microbiota into butyrate, exert anti-fibrotic effects accounting for their protective effect on pulmonary fibrosis. Further investigations indicate that gut microbiome primarily ferments dietary fibers into short chain fatty acids (SCFAs) like butyrate, acetate, and propionate. These metabolites enter the bloodstream and exert anti-inflammatory effects regulating pulmonary immune responses and inhibiting systemic inflammation that drive lung fibrosis (15). Experts suggest a normocaloric high-fiber diet and vitamins could help to counteract dysbiosis-driven inflammation (50).

### Limitations of studies on lung microbiome

Several factors may limit the reliability of studies evaluating the role of lung microbiome in CTD-ILD development. They include: (i) impact of immunosuppressive therapies (i.e., corticosteroids, disease modifying antirheumatic drugs, biologics) on lung microenvironment; (ii) exposure to air pollutants and smoking that may be involved in dysbiosis and fibrosis development; (iii) recurrent infections that may imbalance lung microenvironment (7).

## Conclusion

Research progress has provided new insights for understanding gut and lung dysbiosis in CTD-ILD focusing on clinical lung aspects and therapeutic options. In summary, studies reported in this concise review suggest that microbial dysbiosis could influence disease development and progression. Probiotics and high-fiber diet are promising treatment for CTD-ILD. Future research with longitudinal studies tracking microbiome changes over time will further characterize species that shift in the course of disease to personalize clinical management.
